# Short-Term Space-Use Patterns of Translocated Mojave Desert Tortoise in Southern California

**DOI:** 10.1371/journal.pone.0134250

**Published:** 2015-09-09

**Authors:** Matthew L. Farnsworth, Brett G. Dickson, Luke J. Zachmann, Ericka E. Hegeman, Amanda R. Cangelosi, Thomas G. Jackson, Amanda F. Scheib

**Affiliations:** 1 Conservation Science Partners, 5 Old Town Square, Suite 205, Fort Collins, Colorado, United States of America; 2 Kaweah Biological Consulting, Inc., Three Rivers, California, United States of America; 3 Scheib Biological, Las Vegas, Nevada, United States of America; Smithsonian Conservation Biology Institute, UNITED STATES

## Abstract

Increasingly, renewable energy comprises a larger share of global energy production. Across the western United States, public lands are being developed to support renewable energy production. Where there are conflicts with threatened or endangered species, translocation can be used in an attempt to mitigate negative effects. For the threatened Mojave desert tortoise (*Gopherus agassizii*), we sought to compare habitat- and space-use patterns between short-distance translocated, resident, and control groups. We tested for differences in home range size based on utilization distributions and used linear mixed-effects models to compare space-use intensity, while controlling for demographic and environmental variables. In addition, we examined mean movement distances as well as home range overlap between years and for male and female tortoises in each study group. During the first active season post-translocation, home range size was greater and space-use intensity was lower for translocated tortoises than resident and control groups. These patterns were not present in the second season. In both years, there was no difference in home range size or space-use intensity between control and resident groups. Translocation typically resulted in one active season of questing followed by a second active season characterized by space-use patterns that were indistinguishable from control tortoises. Across both years, the number of times a tortoise was found in a burrow was positively related to greater space-use intensity. Minimizing the time required for translocated tortoises to exhibit patterns similar to non-translocated individuals may have strong implications for conservation by reducing exposure to adverse environmental conditions and predation. With ongoing development, our results can be used to guide future efforts aimed at understanding how translocation strategies influence patterns of animal space use.

## Introduction

Globally, renewable energy generation from all sources, including solar and wind energy, as a proportion of all energy production stands at approximately 19%, has doubled in capacity over the past ten years [1]. Over the next 30–40 years, growth in this sector will increase substantially as world-wide demand for renewable energy continues to grow [2]. Under ‘moderate’ growth projections, renewable energy production is expected to grow to 30–45% of all global energy production by 2050, with ‘high’ growth projections putting the proportion at 50–95% [2]. Across the western United States, regions deemed highly suitable for renewable energy development also tend to be some of the most ecologically sensitive and harbor many species of conservation concern [3, 4]. Currently, federal and state resource agencies are developing broad-scale plans, such as the Desert Renewable Energy Conservation Plan (http://www.drecp.org, accessed November 26, 2014), that simultaneously consider public land, habitat, and species preservation in the face of an expanding footprint of renewable energy infrastructure. In particular, the Mojave desert of southern California (U.S.A.) is expected to experience increased land conversion to accommodate the solar power industry [5, 6].

Recent, rapid growth in the renewable energy sector is due, in part, to concerns about climate change [7], as well as state (California Senate Bill 2X (Simitian) 2011) and federal (Energy Policy Act 2005, American Recovery and Reinvestment Act 2009) regulatory initiatives that incentivize alternative energy production, including solar. Although solar energy may provide the benefit of decreased carbon dioxide emissions and is frequently perceived by the public as ‘clean‘ energy [8], the impacts of solar development on wildlife are poorly understood (but see [9] for a relative ranking of wildlife impacts based on various ‘green energy’ technologies). The construction, maintenance, operation, and decommissioning of utility-scale (i.e., capacity > 20 megawatts) solar energy projects may impact species of conservation concern through habitat loss, degradation, and fragmentation, as well as through displacement of individuals from previously occupied areas or direct mortality [5, 6]. However, in the U.S. and internationally, few of these potential impacts have been quantified in a comprehensive or scientifically rigorous manner [10]. In the vast majority of cases, when translocation is used to help mitigate the potential impacts (e.g., mortality) of energy development projects, and not strictly for conservation purposes (e.g., to augment a declining population), there is a lack of science-based implementation and monitoring to evaluate the effects of translocation [10].

In the western U.S., one species impacted by utility-scale renewable energy development is the federally threatened Mojave desert tortoise (*Gopherus agassizii*) [11]. To offset potential impacts from construction and related human activities, translocation outside of project areas has become a tool used to manage wild tortoises as part of overall mitigation activities [12]. Nevertheless, few published studies have investigated the movement and space-use patterns of translocated tortoises in the Mojave Desert (but see [12, 13, 14]). It has been suggested that altered movement patterns, due to unfamiliarity with resources [15], can potentially put tortoises at greater risk of predation; however two previous studies [12, 16] did not observe this. In addition, altered movement patterns may result in thermal stress, dehydration, and starvation [17], and possibly an increased likelihood of contacting diseased tortoises [18]. Several large-scale utility projects are planning, or have already begun, to translocate tortoises as part of their development plans, yet little is known about the effects of translocation on tortoise space-use patterns considering the risk factors noted above. In the context of ongoing or new energy development projects, an improved understanding of how translocation influences space-use patterns by Mojave desert tortoises is crucial for identifying best management and conservation strategies for this species.

The Ivanpah Solar Electric Generating System (ISEGS) in southern California is presently the largest solar thermal power plant in the world, capable of producing > 390 MW of power. At full capacity, the facility can account for 30% of all solar thermal energy in the U.S and power approximately 140,000 homes per year (http://www.brightsourceenergy.com press release dated February 13, 2014). Beginning in April of 2012, tortoises located within the boundary of the ISEGS project were translocated from an on-site quarantine facility (see [19] for site description and husbandry practices) to an adjacent area known to contain resident tortoises. Here, we contrast these short-distance (typically < 500m) translocations, which were intended to increase the likelihood that tortoises would retain a portion of their putative home range, with previous translocations (e.g., [12]), where tortoises were translocated a greater distance, and possibly outside of their previous home range. Short-distance translocation was intended to determine if it might potentially result in less exposure to the stressors described above, with the ultimate goal of reducing potential negative impacts on tortoise survival. The subsequent monitoring of translocated individuals, as well as individuals in the surrounding population, provides an opportunity to evaluate space-use patterns in a science-based analytical framework, something only a few mitigation-based translocation efforts have engaged in for any species [10]. Additionally, this monitoring establishes critical baseline information on Mojave desert tortoise ecology in a region of rapid energy development.

For the landscape that encompassed the ISEGS and adjacent areas, our principal objective was to evaluate the short-term effect of short-distance translocation on Mojave desert tortoise space-use patterns during portions of the year when tortoises can typically be found above ground (i.e., the ‘active season,’ ranging from approximately the beginning of April to late October). Specifically, we sought to: 1) capture, measure, and monitor tortoises in translocated, resident, and control groups over two active seasons; 2) use radio telemetry data to estimate and compare home range size, home range overlap, and space-use intensity for tortoises in each group; and 3) identify determinants of space use among all groups using information on individual tortoise size and sex, key habitat attributes, and study group identity (translocated, control, and resident).

## Methods

### Study Area

**Fig 1:**
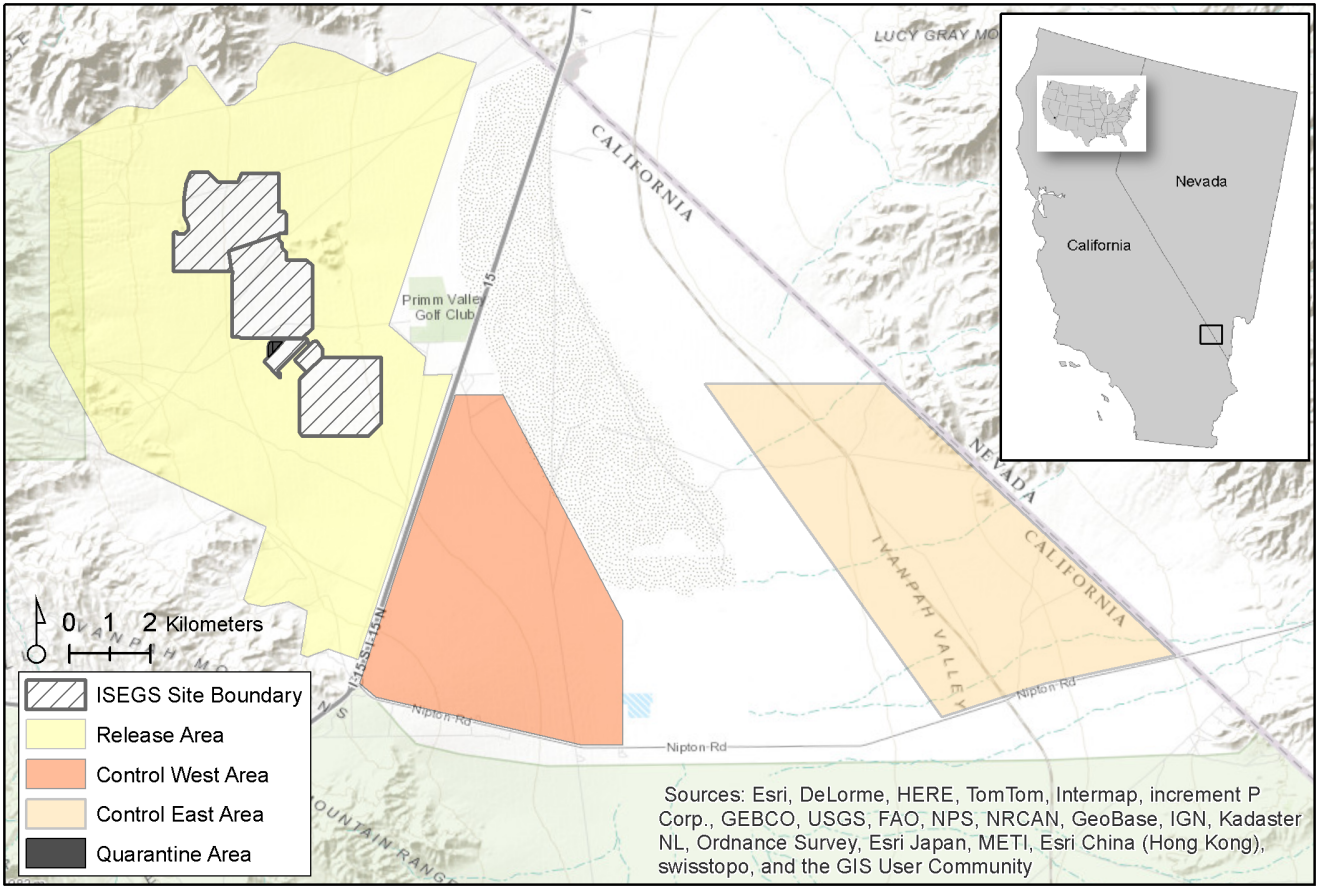
Location of the study area in the Ivanpah Valley of southern California, USA. The Ivanpah Solar Electric Generating System project footprint (white boundary), as well as the locations of the translocated and resident (yellow area), control west (orange), and control east (beige) study groups also are shown.

Our study area was located in the Ivanpah Valley of southern California, approximately 75 km southwest of Las Vegas, NV, in the eastern Mojave Desert (Fig 1). The study site is administered by the U.S. Bureau of Land Management, which approved of the U.S. Fish and Wildlife Service’s plan to monitor all tortoises in this study [20]. The Ivanpah Valley presently encompasses two active renewable energy facilities and one in the development phase, but also is recognized as important for maintaining linkages between Mojave desert tortoise conservation areas in California and Nevada [21]. Monitoring of individual tortoises occurred in three distinct areas (see below) totaling approximately 18,000 ha, including ISEGS. Elevation across the valley ranged from 790 to 1830 m, with topography that consisted of a series of coalescing alluvial fans (or ‘bajadas’), as well as a relatively dense network of braided washes. Both lowland and upland communities consisted of typical Mojave Desert scrub and were dominated by the perennial shrubs creosote bush (*Larrea tridentata*) and white bursage (*Ambrosia dumosa*), although upland communities were typically more diverse. Average annual precipitation was approximately 20.1 cm (1980–2010; Global Historical Climatology Network station USC00267369, Searchlight, NV), however, in recent years, total annual precipitation at that station was recorded as 17.9 cm (2011), 12.6 cm (2012), and 14.7 cm (2013).

### Study Groups

We established four unique study groups in three distinct areas (Fig 1) for monitoring and analysis: translocated, resident, control west, and control east. Beginning in October of 2010, U.S. Fish and Wildlife Service-authorized biologists located and captured tortoises within the ISEGS boundary and placed them in quarantine pens established on the project site to ensure that none of the individuals to be translocated exhibited clinical signs of disease. All tortoise handling and tracking procedures were explicitly mandated and permitted by the U.S. Fish and Wildlife Service [19], the primary entity responsible for managing the Mojave desert tortoise. In the spring of 2011, surveys were conducted to locate, measure, and, as appropriate, radio-tag tortoises in the control east area (see below). An identical effort was conducted in the fall of 2011 to establish the control west and resident groups. The two control groups were established as a baseline for comparison with translocated tortoises. The resident group was established to examine the potential influence translocated tortoises might have on space-use patterns of tortoises with an established home range. As specified by the Biological Opinion [20], ‘translocated’ tortoises in our study were defined as those individuals moved from quarantine pens to a release area (8798 ha) immediately adjacent to ISEGS (1368 ha) because they had grown to have a midline carapace length (MCL) of at least 120 mm by April of 2012 when the translocation event occurred. All tortoises remaining in the quarantine pens after this date were less than 120 mm and scheduled for a future translocation event. All translocated tortoises in our study were captured inside the project footprint and were ≤ 500 meters of the project boundary. In addition, The Biological Opinion mandated that the release site for each translocated animal be ≤ 500 m from their median location prior to being removed from the project site, and placed into the quarantine pens during clearance surveys. Finally, each tortoise was tracked prior to being removed and taken to the quarantine pens, thus we had information allowing us to identify at least a portion of each individual’s previous home range.

Because the project footprint was enclosed behind tortoise-proof fencing, it was not possible for translocated tortoises to return to portions of their previous home range that may have fallen within the project footprint. The release area included individuals we refer to as ‘residents.’ Two ‘control’ groups (control west [3560 ha] and control east [4220 ha]) were established outside the project footprint in areas encompassing a range of habitat attributes similar to those observed within and immediately adjacent to the ISEGS site prior to construction. Specifically, we considered (and refer to) two distinct control groups because they 1) were separated by a mostly impermeable railroad line and the southern extent of Ivanpah Dry Lake and 2) each occupied areas with distinct topographic (control west = lowland, control east = upland) and vegetation characteristics. In addition, we wanted to understand how the two different vegetation communities in the control sites might be related to translocated tortoise movement patterns. We radio-tagged tortoises in the resident and control groups such that the distributions of sexes and sizes were as similar as possible to the translocated group.

### Field Data

We fitted individuals in each study group with very high frequency (VHF) radio transmitters (Holohil Systems Ltd., Ontario, Canada) using the method described in [22]. We located (i.e., encountered) all tortoises on an approximately weekly basis during the 2012 and 2013 active seasons, between the hours of 0600 and 1800, independent of weather conditions. During each encounter, we recorded Universal Transverse Mercator (UTM) coordinates and information about whether or not an individual was in a burrow. During bi-annual (May and September) health assessments we also recorded MCL, which was highly correlated with weight. In addition, the sex of each individual was classified as male, female, or unknown. Tortoises classified as unknown were overwhelmingly sub-adults who were too young to be identified as male or female. Studies in the region have identified that differential space-use patterns can occur between the sexes, and for sub-adult and juvenile versus adult tortoises [13, 23].

### Space-Use Estimation

Within each active season and study group, we derived estimates of space use (*sensu* [24, 25]) for each tortoise using all encounters obtained in that season by taking a kernel density estimation approach [26]. We excluded individuals with < 25 encounters during an active season to balance selecting an appropriate minimum number of encounters with censoring of individuals from the dataset. We used the Geospatial Modeling Environment (GME; [27]) in a geographic information system (GIS; ArcGIS version 10.1, Esri, Redlands, CA, U.S.A.) to calculate 95% fixed kernel density estimates (KDEs) and resultant utilization distributions (UDs) at a 30-m pixel resolution. We also refer to the area under the UD as the home range of an individual. The GME used the R software environment (version 2.14, R Development Core Team 2011) to select the optimal bandwidth value for each UD based on a maximum likelihood estimator [28]. The KDE approximates a three-dimensional probability density function, exhibiting higher probability mass (i.e., space-use intensity) in portions of the surface having a greater density of encounters. Because tortoise encounters occurred on a weekly basis, we were not concerned about potential issues of spatial or temporal serial autocorrelation. We used the GIS to relate individual encounters to KDE values (i.e., UD pixels) by intersecting each UD with the locations used to derive it.

To complement the above analyses, we used the encounter data to create a proxy for movement based on distance and elapsed time between encounters. First, we calculated the Euclidean distance between consecutive encounters for each tortoise using UTM coordinates. We then scaled this distance by the number of days between consecutive encounters to obtain a rate (m/day). In a few instances, consecutive encounters occurred within a single day and those records (2.8% and 0.4% of the 2012 and 2013 records, respectively) were removed. Finally, we computed the mean of the rate (m/day) for each tortoise, and in all study groups for each active season, and then graphically evaluated between-group differences using density plots. While we recognized that distance between encounter events and distance traveled are fundamentally different variables, we considered the time-scaled distance between encounters to be a coarse proxy for general movement patterns and complementary to the space-use analyses described above.

Finally, we calculated percentages of home range overlap based on the UDs for resident and translocated tortoises in 2012 and 2013 using the kernel method described in [29]. Briefly, for each pair of individuals in each group, we calculated percentage overlap, *O*, as

$$O=\frac{A_{1,2}}{\left(A_{1}+A_{2}\right)-A_{1,2}},$$

where *A*_1_ is the UD area of tortoise 1, *A*_2_ is the UD area of tortoise 2, and *A*_1,2_ is the area of overlap between the two UDs. We then calculated a mean overlap value to make comparisons within and between study groups in each year, as well as within and between male and female tortoises in the groups.

### Environmental Variables

As patterns of space use among study groups are likely driven by life-history needs, we identified and derived three key environmental variables for our statistical models. First, given the importance of burrow use to many facets of desert tortoise life history [30, 31], each encounter was classified as either finding the tortoise in a burrow or not, based on field observations. Second, because tortoises rely heavily on shrub cover for thermoregulation and on washes as movement corridors and for substrate used to dig burrows [32, 33], we derived spatially explicit models (i.e., data layers) of shrub and wash density using 1-m resolution, four-band color-infrared digital ortho quarter quad (CIR DOQQ) imagery acquired from the USDA National Agricultural Imagery Program (July 2009) and a Random Forest method for classification [34]. Within the 15 CIR DOQQ tiles (each a 3.75 × 3.75-minute quarter quadrangle, plus a 300-m buffer) that overlapped our study, we used 120,000 random points to identify several land cover classes, including shrub and wash classes. Red and near-infrared bands were used to derive Normalized Difference Vegetation Index (NDVI) and all four bands, along with NDVI, were filtered with Gaussian, Sobel, and Laplacian neighborhood convolution filters to provide spatial and textural context and to help discriminate between classes. We used the ‘randomForest’ package [35] in R to implement our regression tree model and classify land cover types, and used Cohen’s kappa statistic to evaluate model accuracy [36]. For the wash class, our initial model result was refined using a circuit theory-based estimate of connectivity (i.e., spatial contiguity; [37]) across the study landscape. We used Circuitscape software (version 3.5, www.circuitscape.org) to generate a final wash model. Wash and shrub classification accuracy was 88.4% and 96.0% with associated Cohen’s kappa values of 0.682 and 0.891, respectively. We calculated mean wash density and shrub density by resampling the original layers at a 30-m resolution and related these outputs to the value of space-use intensity at each encounter location.

### Statistical Modeling

For both active seasons, we calculated the distance each encounter location was from the project footprint for the translocated group and compared the distribution of distances between 2012 and 2013. To compare home range estimates among the study groups and sex classes, we fit linear mixed-effects models to the log-transformed UDs, with either study group or sex class as a fixed effect and individual as a random effect using the ‘nlme’ package [38] in R. We then conducted multiple comparisons tests (α = 0.05) using the ‘multcomp’ [39] package in R. We log-transformed the UDs to meet the parametric assumption of our statistical tests.

Within an information-theoretic framework, we used a linear mixed-effects model structure and multi-model inference [40] to estimate and compare the determinants of space-use intensity, given the log-transformed KDE values and explanatory variables (i.e., fixed effects) described above. We treated study group (translocated, resident, control east, control west), sex (male, female, unknown), active season year (2012 and 2013), MCL, shrub density, and wash density as fixed effects. In addition to the linear terms for shrub density and wash density, we included a quadratic term to examine the influence of non-linearity in these environmental variables on space-use intensity. We also included a year × study group interaction effect to determine if space-use intensity varied by study group and active season relative to the control west group we selected as a baseline for comparisons. Each individual tortoise was treated as a subject-level random effect to account for heterogeneity in space-use intensity among individuals [41]. We accounted for within-tortoise temporal correlation, based on Julian date of encounter, by specifying a powered exponential covariance structure. To account for any residual spatial autocorrelation in the tortoise encounters, we computed the variance-covariance matrix of the fixed-effects with the asymptotically consistent sandwich estimator [42]. Prior to modeling, we standardized all continuous variables. We calculated variance inflation factors to investigate collinearity among these variables, although no value > 2.5 was observed, thus we were able to include all continuous variables in our model.

Given all of the above variables, we formulated a single, full model of space-use intensity by tortoises and used Akaike’s Information Criterion (AIC) for inference [40]. We used an intercept-only model (including the random effects) and the difference in AIC (ΔAIC) values to evaluate how well our full model approximated the data [43]. We considered a model with ΔAIC value > 10 AIC units from (lower than) the intercept-only model to represent a good approximation of the data [43]. To compare the relative strength of association between demographic and environmental variables (*j*) and the log-KDE values, we used multi-model inference (i.e., all-subsets modeling) to compute model-averaged regression coefficients, ($\tilde{\bar{\beta }}$),unconditional standard errors (SEs), cumulative AIC weights of evidence (*w*_+_(*j*)) as a measure of variable importance, and 95% confidence intervals. Our interpretation of the explanatory power of the regression coefficients was guided by two measures: 1) the weights of evidence, ranging from 0 to 1.0, where higher weights indicate greater importance; and 2) the 95% confidence interval for each regression coefficient. We implemented all models with PROC MIXED in SAS (v.9.3; SAS Institute 2012) and the R statistical environment.

## Results

Between April 2012 and October 2013, we monitored 308 individual tortoises relocated ≥ 25 times in a given year within the translocated (*n* = 54), resident (*n* = 118), control west (*n* = 105), and control east (*n* = 31) study groups (Table 1). Of these individuals, 148 were male, 112 were female, and the sex of 48 individuals could not be determined at the time of capture (Table 2). Average MCL was 219.0 mm (SD = 46.2). Adult tortoises whose sex could be identified had MCL values ranging from 165 to 315 mm. Tortoises classified as an unknown sex (i.e., juvenile or sub-adults) had MCL values that ranged from 67 to 165 mm.Across active seasons and groups, we obtained an average of 68.9 encounters (SD = 11.4) per individual with 63.5% of the encounters indicating tortoises were in burrows.

**Table 1:** Number and composition of individual tortoises in each study group during the 2012 and 2013 active seasons, partitioned by average midline carapace length (MCL, in millimeters), MCL standard error (SE_MCL_), average utilization distribution (UD, in hectares) and UD standard error (SE_UD_).

Group	Count	MCL	SE_MCL_	UD^1^	SE_UD_
*2012*					
Control East	31	214	36	24	33
Control West	104	211	53	29	42
Resident	114	226	45	46	77
Translocated	54	215	39	97	130
*2013*					
Control East	28	224	31	33	44
Control West	103	215	52	36	62
Resident	112	229	46	50	71
Translocated	46	223	32	100	430

^1^The UD is defined as the area encompassed by the 95% kernel density estimate for each tortoise.

**Table 2:** Number and composition of individual tortoises by sex during the 2012 and 2013 active seasons, partitioned by average midline carapace length (MCL, in millimeters), MCL standard error (SE_MCL_), average utilization distribution area (UD, in hectares) and UD standard error (SE_UD_).

Sex^1^	Count	MCL	SE_MCL_	UD^2^	SE_UD_
2012					
Female	112	220	19	31	62
Male	146	244	29	64	90
Unknown	43	131	31	32	77
*2013*					
Female	109	223	17	21	32
Male	144	248	26	88	260
Unknown	38	128	31	11	20

^1^Tortoises classified as sex = Unknown were overwhelmingly sub-adults too small to be identified as male or female.

^2^The UD is defined as the area encompassed by the 95% kernel density estimate for each tortoise.

The average distance from the boundary of the project footprint to where translocated tortoises were encountered in 2012 was 357 m (SD = 456) and in 2013 was 505 m (SD = 620), which was significantly (*Z* = -7.53, *p* ≤ 0.001) closer to the project footprint in 2012 than in 2013. For the 2012 and 2013 active seasons, the average UD area was 47.9 ha (SD = 86.0) and 51.2 ha (SD = 183.7), respectively. Considering the average, log-transformed UD of each group, we observed significantly (*Z* always > 3.38, adjusted *p* always ≤ 0.001) larger UD areas for translocated tortoises when compared to each of the other groups in 2012 (Table 3). No significant (*Z* always < 0.85, adjusted *p* always ≥ 0.83) differences were detected between any groups in 2013. In both years, males had significantly (*Z*_2012_ = 5.58, *p*_2012_ ≤ 0.001; *Z*_2013_ = 9.08, *p*_2013_ ≤ 0.001) larger UD areas than females. Tortoises of unknown sex had significantly (*Z*_2012_ = -8.48, *p*_2012_ ≤ 0.001; *Z*_2013_ = -12.20, *p*_2013_ ≤ 0.001) smaller UD areas than males and significantly (*Z*_2012_ = -4.29, *p*_2012_ ≤ 0.001; *Z*_2013_ = -5.69, *p*_2013_ ≤ 0.001) smaller UD areas than females (Table 4).

**Table 3:** Summary of multiple comparisons *Z-*tests of pairwise differences (Estimate) in mean log-UD (i.e., log-hectares encompassed by the 95% kernel density estimate) among the four tortoise study groups, including standard errors (SE), test statistics (*z*-value), and adjusted *p*-values for the 2012 and 2013 active seasons.

Comparison	Estimate	SE	*Z-*value	*p*-value
*2012*				
TR-CW	1.29	0.24	5.41	< 0.001
TR-CE	1.25	0.32	3.93	< 0.001
TR-RE	0.79	0.24	3.38	< 0.001
RE-CW	0.49	0.19	2.56	0.05
RE-CE	0.46	0.29	1.60	0.37
CE-CW	-0.03	0.29	-0.12	1.00
*2013*				
TR-CW	0.20	0.24	0.85	0.83
TR-CE	-0.05	0.32	-0.16	1.00
TR-RE	-0.12	0.23	-0.51	0.96
RE-CW	0.32	0.18	1.77	0.28
RE-CE	0.07	0.28	0.24	1.00
CE-CW	0.25	0.28	0.89	0.81

CW = control west; CE = control east; RE = resident; TR = translocated. Significant differences were detected between the translocated group and each of other groups in the 2012 active season; no other statistically significant differences were detected.

**Table 4:** Summary of multiple comparisons *Z-*tests of pairwise differences (Estimate) in mean log-UD (log-hectares encompassed by the 95% kernel density estimate) among the sexes, including standard errors (SE), test statistics (*z*-value), and adjusted *p*-values for the 2012 and 2013 active seasons.

Comparison	Estimate	SE	*Z*-value	*p*-value
*2012*				
M-F	0.93	0.17	5.58	< 0.001
U-F	-1.02	0.24	-4.29	< 0.001
U-M	-1.94	0.23	-8.48	< 0.001
*2013*				
M-F	1.21	0.13	9.08	< 0.001
U-F	-1.12	0.20	-5.69	< 0.001
U-M	-2.33	0.19	-12.20	< 0.001

F = Female; M = Male; U = Unknown.

**Fig 2:**
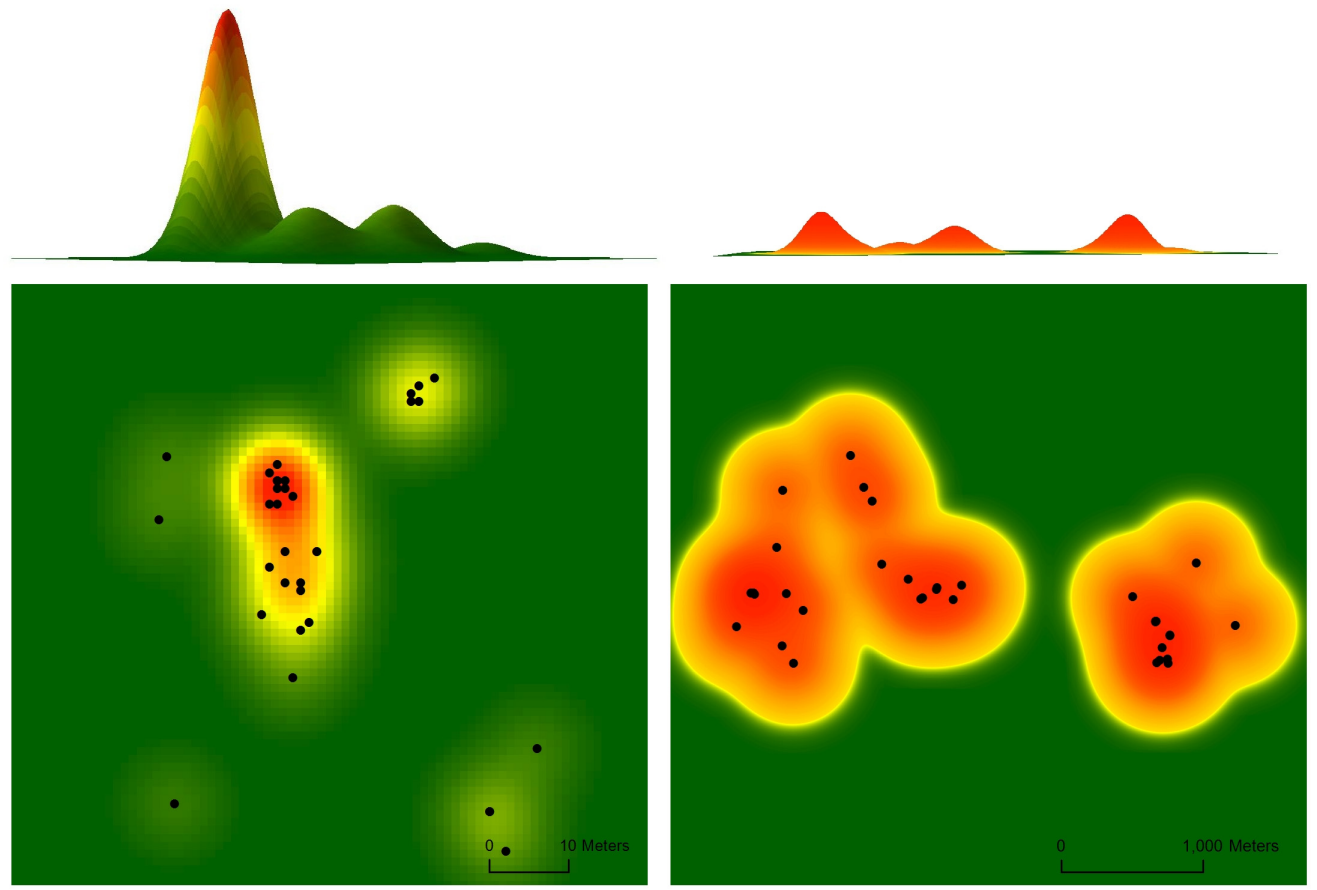
Example utilization distribution (spatial extent) and 95% kernel density estimate (height and coloration), as a metric of space-use intensity, for a control west (left two panels) and translocated (right two panels) tortoise monitored during the 2012 active season (approximately April through October). The control west individuals exhibited greater space-use intensity than translocated tortoises, which is reflected in the higher peaks and more limited spatial extent associated with the control west tortoise’s utilization distribution.

**Fig 3:**
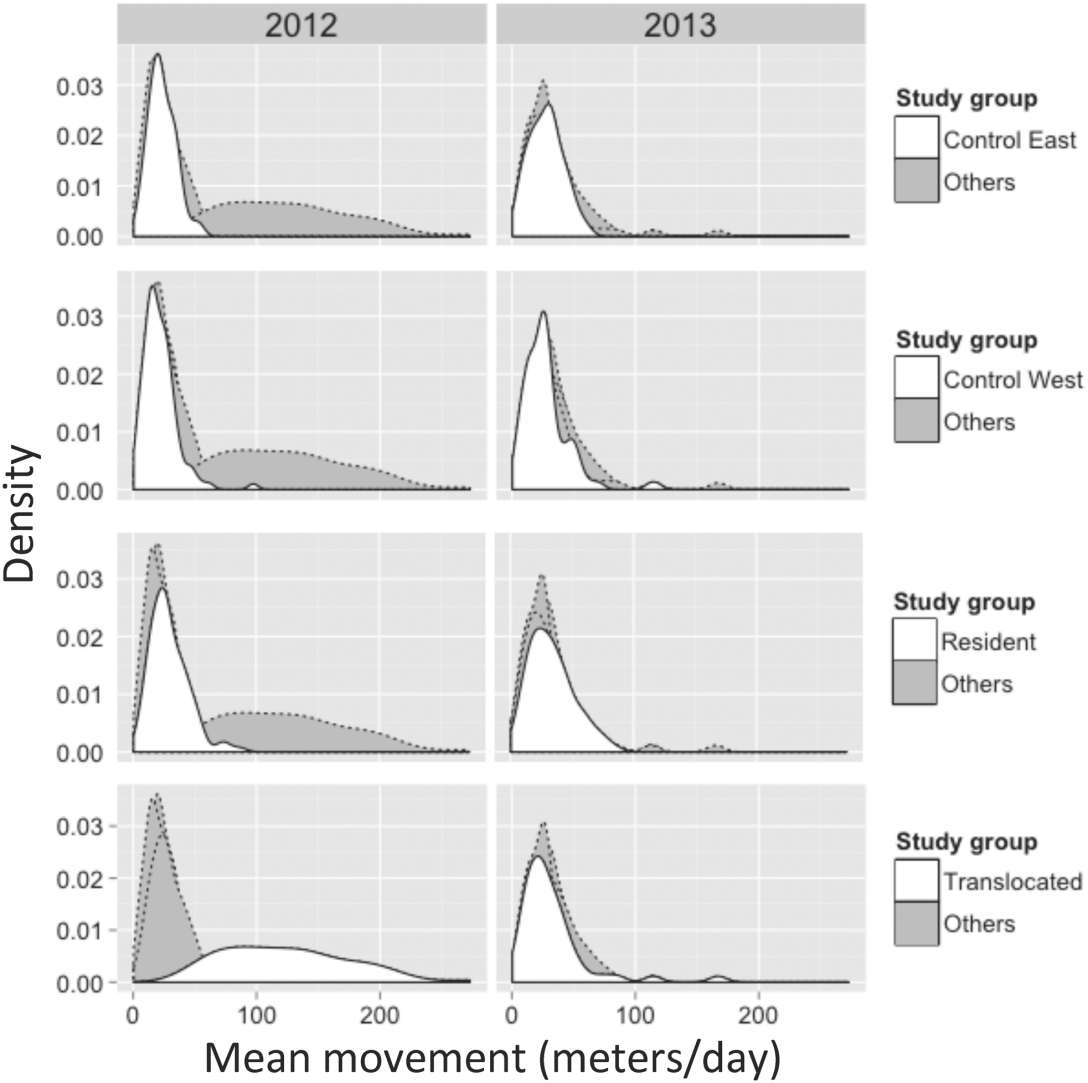
Density plots of the distribution of time-scaled distances (in meters; adjusted for days between subsequent encounters) for tortoises monitored during the 2012 (left column) and 2013 (right column) active seasons (approximately April through October). The distributions shown in white in each row represent one of the four study groups (indicated by the legend for each row) while shaded distributions represent all other groups. Note the difference in distributions between the 2012 translocated tortoises (lower left panel) versus all other study groups in both years.

The 95% KDE surfaces (Fig 2) extracted from the UD for two tortoises in our 2012 encounter data provide an example of a control tortoise with a relatively high (left two panels) space-use intensity and a translocated tortoise having a relatively low (right two panels) space-use intensity. Higher portions of the surface correspond to increased space-use intensity. Based on smoothed density plots, mean time-scaled distances (m/day) between consecutive encounters were higher for the translocated group than for all other groups in 2012 (Fig 3). This pattern was not observed in 2013, when all study groups appeared to have similar time-scaled distances between subsequent encounters.

**Fig 4:**
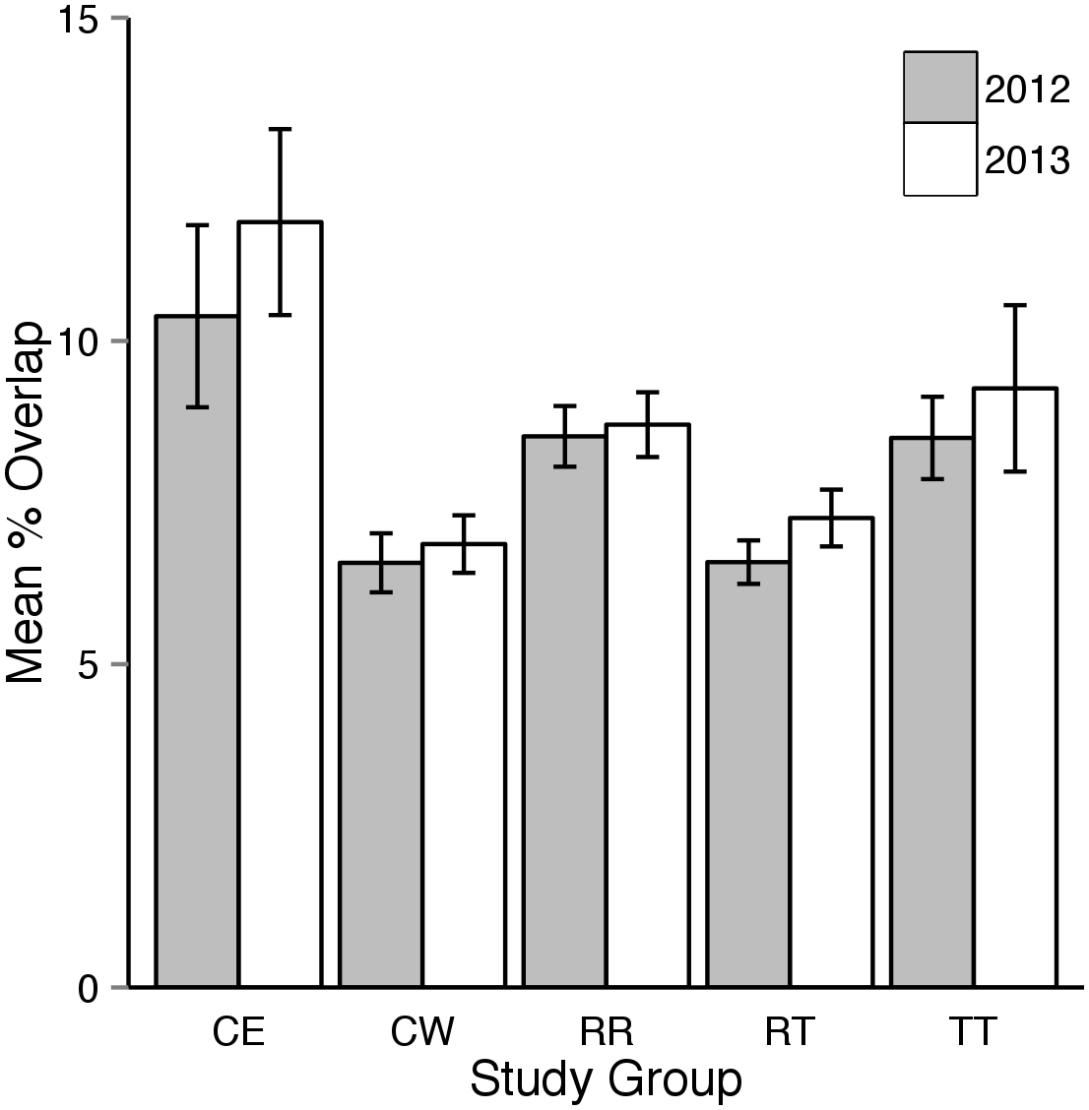
Estimates of mean percentage overlap of 95% utilization distributions between resident and translocated tortoises monitored during the 2012 (grey bars) and 2013 (white bars) active seasons (approximately April through October). TT = overlap between tortoises in the translocated study group, RR = overlap between tortoises in the resident study group, and TR = overlap between tortoises in the translocated study group with those in the resident group. Note that overlap between translocated study group individuals was more than twice as great in 2012 compared to 2013.

**Fig 5:**
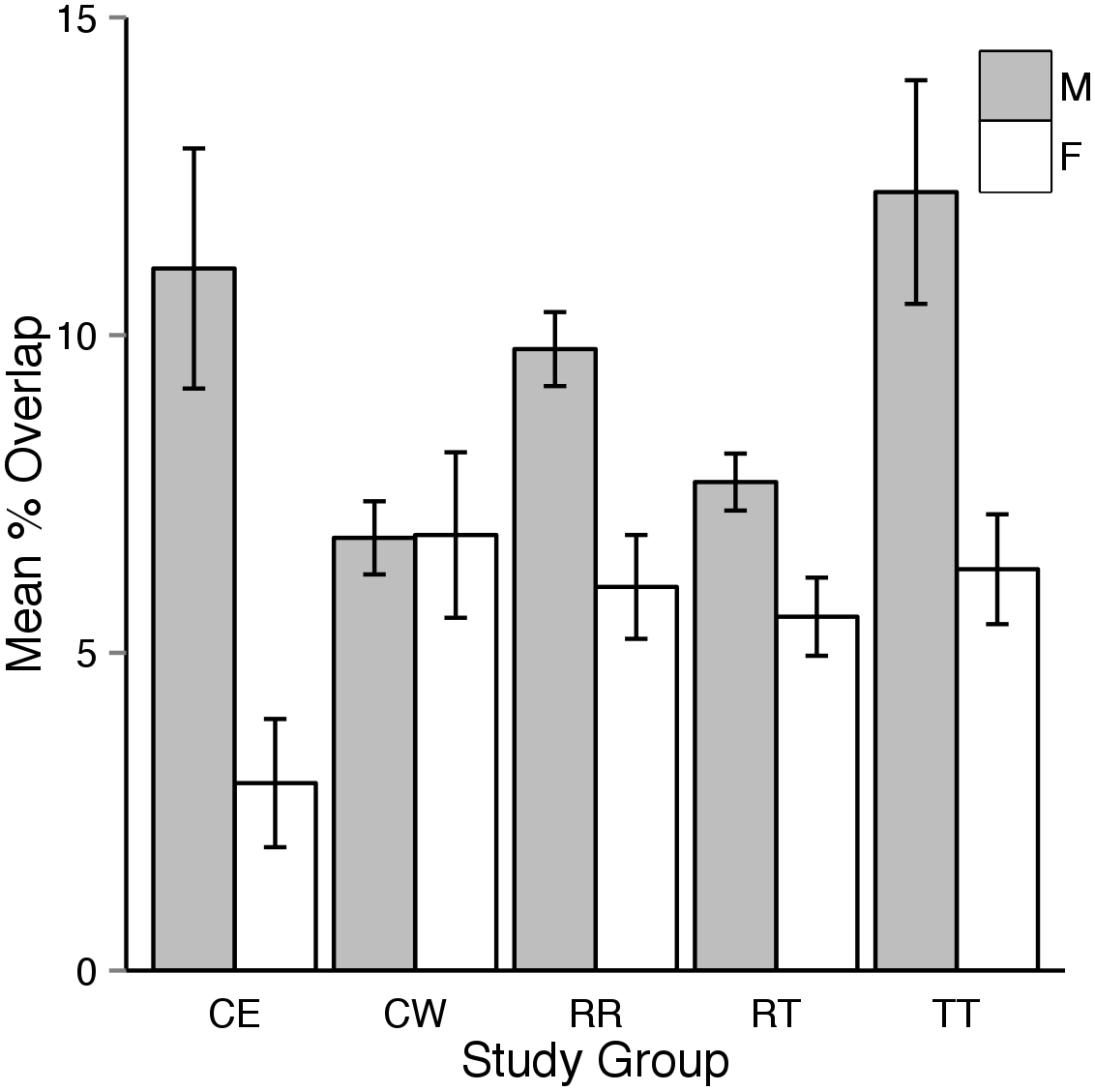
Estimates of mean percentage overlap of 95% utilization distributions between resident and translocated male and female tortoises monitored during the 2012 and 2013 active seasons combined (approximately April through October). TT = overlap between tortoises in the translocated study group, RR = overlap between tortoises in the resident study group, and TR = overlap between tortoises in the translocated study group with those in the resident group. Grey bars represent overlap of males with other males and white bars show the percentage of overlap between females. Note that males in all comparisons had greater overlap than did females and those tortoises in the translocated study group had greater overlap for both sexes than in the other comparisons.

Mean percentage overlap was slightly greater in 2013 compared to 2012, however the confidence intervals overlapped in all comparisons (Fig 4). Males typically had greater overlap with each other than did females with other female tortoises (Fig 5).

The ΔAIC value of our full model of space-use intensity, based on log-KDE as the response variable, was approximately 1000 AIC units less than the intercept-only model, suggesting an excellent approximation of the data. The variables translocated (*w*_+_(*j*) = 1.0), translocated × year (1.0), burrow (1.0), sex = male (1.0), sex = unknown (0.99), and shrub density (1.0) indicated the highest relative importance in our model of tortoise space-use intensity (Table 5). With the exception of the variable year, for which *w*_+_(*j*) = 0.79, the remaining variables all had weights ≤ 0.66, suggesting a smaller influence on tortoise space-use intensity relative to other variables in the model.

**Table 5:** Model-averaged parameter estimates ($\tilde{\bar{\beta }}$) unconditional standard errors (SE), 95% confidence intervals (CI), and cumulative Akaike’s Information Criterion weights (w_+_(*j*)) for all variables used to model space-use intensity in the combined 2012 and 2013 active seasons.

Variable	$\tilde{\bar{\beta }}$	SE	CI	w_+_(*j*)
Intercept	2.74	0.11	(2.52, 2.96)	NA
Translocated	-1.13	0.21	(-1.53, -0.73)	1.00
Resident	-0.09	0.12	(-0.32, 0.15)	0.52
Control East	0.05	0.15	(-0.24, 0.34)	0.35
Year	-0.19	0.14	(-0.45, 0.08)	0.79
Year × Translocated	0.95	0.28	(0.41, 1.50)	1.00
Year × Resident	0.007	0.13	(-0.25, 0.26)	0.36
Year × Control East	-0.16	0.25	(-0.66, 0.34)	0.46
Sex Male	-1.03	0.10	(-1.21, -0.84)	1.00
Sex Unknown	0.76	0.22	(0.33, 1.20)	0.99
Burrow	0.57	0.02	(0.53, 0.60)	1.00
Shrub	-0.04	0.02	(-0.09, 0.005)	1.00
Shrub^2^	-0.0006	0.008	(-0.02, 0.02)	0.28
Wash	0.005	0.01	(-0.02, 0.03)	0.43
Wash^2^	-0.005	0.008	(-0.02, 0.01)	0.65
MCLavg	0.03	0.07	(-0.11, 0.17)	0.37

MCLavg = average midline carapace length in each year. In our design matrix, the categorical variables were specified such that control west, female, and 2012 were the baseline contrasts used for comparison in the study group, sex, and year categories, respectively. Note the negative effect for translocated and positive effect for year × translocated, suggesting that space-use intensity increased for translocated tortoises in 2013 relative to 2012.

The parameter estimate for the variable translocated was negative, with a confidence interval that did not include zero (Table 5). This pattern was not observed in the control east or resident study groups, whose confidence intervals also included zero. The year × translocated interaction term was positive, indicating an increase in the concentration of space use in 2013. The year × study group interaction for the resident and control east groups had confidence intervals that included zero.

When a tortoise was classified as being encountered in a burrow, its estimated space-use intensity increased relative to when encounters were classified as non-burrow (Table 5). Males exhibited more diffuse space-use patterns than females, and animals classified as an unknown sex, which were overwhelmingly sub-adults too small to be differentiated, showed space-use patterns that were more concentrated on average than females. For the shrub density variable, there was a negative relationship with increasing space-use intensity. We did not detect a clear relationship between space-use intensity and wash density or MCL.

## Discussion

Globally, translocation is increasing as a tool selected by land managers when development-related conflicts occur with species of concern [44]. In the past fifteen years alone, mitigation-driven translocations for multiple species have increased sharply [45]. Nevertheless, many countries do not require monitoring of translocated individuals or do not make monitoring data easily accessible [10]. Our study differs in that a key requirement for developing the ISEGS project was that monitoring, as one component of a comprehensive and science-based program, be conducted for each of the study groups [20]. Although translocation (over any distance) is a relatively new tool that is increasingly being used when conflicts occur with land use or development, including construction of renewable energy facilities [12], managers need information that will help them evaluate the short- and long-term impacts of development on tortoise populations. Results from our study can help fill the need for information and identify key information gaps.

Our investigation of translocation as a mitigation tool differs from previous translocation studies in at least two fundamental ways. First, we used a short-distance translocation approach for tortoises in our study. Second, the metrics we chose to examine were associated with home range size (UDs) and space-use intensity (KDEs), which were conditioned on all encounter data, rather than a single-value summary of the data, such as the maximum measured displacement across an entire year post-translocation. Further, we believe the UD- and KDE-based measures we generated provide deeper insight into tortoise space-use patterns than do summary statistics based solely on commonly used Euclidian distance-based metrics. Because kernel estimators identify spatially explicit space-use patterns on the landscape, they can provide a more meaningful interpretation of variations in behavior among individuals and study groups. In addition, kernel density estimates can easily be tied back to habitat features, thereby linking the process of space-use intensity to a meaningful set of predictor variables. Finally, the surface that results from kernel density estimation can be treated as a repeatable and quantitative measure of an individual’s home range (i.e., the UD).

The home range of the Mojave desert tortoise has been described as a network of burrows connected by travel corridors of varying dimension [46, 47]. During the active season of 2012, the patterns of space use that we observed indicated a process in which translocated tortoises were initially questing for a new home range and resource base (e.g., burrow system, forage, cover). In the following active season, this same group behaved in a manner that increased their space-use intensities and decreased their home range size, resembling the patterns exhibited by non-translocated tortoises. This result suggests that tortoises may require between one and two active seasons to reestablish a burrow network after being translocated a short distance away—but possibly still within a portion of—their former home range. A previous study on Mojave desert tortoises that were translocated entirely out of their putative home range (i.e., using long-distance translocation protocols) suggested a pattern in which tortoises took up to three years to exhibit patterns of space use (based on straight-line distance moved) that were statistically similar to a resident group [13]. In another study [23], a positive association was observed between core home range area, defined as the 50% minimum convex polygon, and the number of burrows used. It is possible that translocated tortoises in our study also relied on a more extensive network of burrows than did the resident and control groups, though we lacked high-resolution data on the spatial extent of burrow use.

Although our study was conducted over two years when rainfall in the Mojave Desert was above average, this region has been in the midst of a long-term drought [48] and more severe drought conditions could become the norm, which will likely have substantial influence over tortoise space-use patterns. During drought years, tortoises have been known to reduce the number of burrows used, as well as increase their space-use intensity and have smaller home ranges relative to non-drought years [47, 49]. This observation further supports the notion that minimizing the time a tortoise spends questing for a new burrow network is crucial, particularly if the translocation takes place during, or immediately preceding, periods of drought. If short-distance translocation aimed at maintaining a portion of an individual’s previous burrow network reduces the number of active seasons required to reestablish an extensive burrow network, then this approach may become the preferred strategy in future translocations associated with development activities, including renewable energy projects.

Because tortoise space-use patterns are likely shaped by a combination of environmental and demographic characteristics, we included several variables in our model that we hypothesized were strong determinants of space-use intensity. When tortoises were classified as being located in a burrow, estimated space-use intensity increased. This is not surprising since—in some parts of their range—individual desert tortoises can spend up to 98% of their time in burrows or pallets [50]. Further, males had more diffuse space-use patterns than females, while individuals of an unknown sex, who were classified overwhelmingly as sub-adults, had more concentrated space use relative to females, consistent with previously reported patterns [23, 51]. The main effect of shrub density, which was negative, suggested that as shrub density increased, space-use intensity decreased. Because the detectable canopies of dominant shrub species (*A*. *dumosa* and *L*. *tridentata*) at our study site is often < 1 m^2^, a causal relationship could not be established, given the aerial imagery we used. Thus, gaining a more precise, mechanistic understanding of the relationship between tortoise space use and shrub density will require an estimate of shrub density that is based on higher resolution data. Consistent with an earlier finding [23], MCL was not a strong predictor of space-use intensity. The lack of correspondence between body size and space-use intensity that we observed was likely due to the use of a subject-level random effect in our model structure. Moreover, because the sex variable (a fixed effect) was able to capture differences in space-use intensity among males, females, and tortoises of an unknown sex, the sex category performed as a better predictor of space-use intensity than MCL. Finally, although we did not detect a relationship between tortoise space-use intensity and wash density, other metrics describing wash characteristics, such as the degree of incision [52], could be more meaningful, data permitting.

In the context of increasing human development and its anticipated impacts on sensitive populations of wildlife in the southwestern U.S. [3, 4], effective animal conservation and management actions will require new, but relatively untested approaches, including rigorous monitoring of sensitive species affected by mitigation-based translocation [10]. In the study described in [12], which was approximately 75 km from our study site, long-distance translocated tortoises that had been moved approximately 32 km from their holding facility exhibited movement patterns in their second year post-translocation that were not statistically different from resident animals. Instead of UD or KDE metrics, these researchers used straight-line distance metrics (e.g., cumulative distance or longest distance moved) for inference. In a separate study [15], researchers experimentally manipulated translocation distances to identify short-term homing by tortoises. They found that as translocation distances increased, tortoises were less likely to attempt to return to their previous home range.

Although short-distance translocation may be advantageous in preserving a portion of an individuals previous home range, it also may result in a stronger homing instinct resulting in greater exposure to potential threats (e.g., by ‘fence pacing‘ when prevented from returning to previous portions of a home range). In our study, we found that in the first year post-translocation, telemetry encounters for translocated tortoises were significantly closer to the project boundary than in the second year. In several cases, tortoises remained relatively close to the project boundary for several months, but moved further away from the boundary as fall approached. In the second year, these tortoises did not appear to attempt to home back to their previous home range. Thus, the effect of short- versus long-distance translocation on movement patterns, and over relatively short time scales, remains a key area for new research as managers seek to understand the impacts of and trade-offs between mitigation strategies.

Translocation-driven changes in the behavioral patterns of the Mojave desert tortoise could increase their exposure to numerous stressors, such as altered thermalregulatory responses, or to direct mortality due to increased predation risk or exposure to diseased resident tortoises. Indeed, identifying translocation strategies that reduce the time a tortoise is exposed to potentially lethal conditions is critical as renewable energy projects in the Mojave Desert continue to expand into the public lands that also provide important tortoise habitat. Because so few studies have examined the effects of translocation on tortoise movement patterns over multiple years, it remains unclear if short-distance translocation reduces the length of time individuals are exposed to various stressors. Results from our and other recent studies should be used to guide future research aimed at understanding how different translocation strategies influence patterns of Mojave desert tortoise space use and—ultimately—contribute to the persistence, conservation, and management of a species confronting an increasing public demand for renewable energy.
